# Breast cancer humoral immune response: involvement of Lewis y through the detection of circulating immune complexes and association with Mucin 1 (MUC1)

**DOI:** 10.1186/1756-9966-28-121

**Published:** 2009-08-28

**Authors:** Marina Isla Larrain, Sandra Demichelis, Marina Crespo, Ezequiel Lacunza, Alberto Barbera, Aldo Cretón, Francisco Terrier, Amada Segal-Eiras, María Virginia Croce

**Affiliations:** 1 Centre for Basic and Applied Immunological Research, Faculty of Medical Sciences, National University of La Plata. Calle 60 y 120, 1900, La Plata, Buenos Aires, Argentina; 2 Breast Foundation, Calle 7 y 42, 1900, La Plata, Buenos Aires, Argentina; 3 Faculty of Natural Sciences, National University of La Plata. Calle 60 y 122, 1900, La Plata, Buenos Aires, Argentina

## Abstract

**Background:**

In cancer patients, MUC1 glycoprotein may carry Lewis y which could be involved in immune response. Purposes: 1- to evaluate the presence of Lewis y and MUC1 in circulating immune complexes (Lewis y/CIC and MUC1/CIC, respectively) and their correlation; 2- to analyze the possible presence of Lewis y in carbohydrate chains of tumoral MUC1 glycoprotein and 3- to correlate serum and tissue parameters considered.

**Methods:**

Pretreatment serum and tissue breast samples from 76 adenocarcinoma, 34 benign and 36 normal specimens were analyzed. Anti-MUC1 and anti-Lewis y MAbs were employed. To detect Lewis y/CIC and MUC1/CIC, ELISA tests were developed; serum samples containing MUC1 were previously selected by Cancer Associated Serum Antigen (CASA). Immunoprecipitation (IP) was performed in 9 malignant, benign and normal samples and analyzed by SDS-PAGE and Western blot. Lewis y and MUC1 expression was studied by immunohistochemistry (IHC). Statistical analysis was performed employing principal component analysis (PCA), ANOVA, Tukey HSD, Chi square test and classical correlation (p < 0.05).

**Results:**

By ELISA, Lewis y/IgM/CIC levels showed statistically significant differences between breast cancer versus benign and normal samples; mean ± SD values expressed in OD units were: 0.525 ± 0.304; 0.968 ± 0.482 and 0.928 ± 0.447, for breast cancer, benign disease and normal samples, respectively, p < 0.05. Lewis y/IgG/CIC did not show any statistically significant difference. MUC1/IgM/CIC correlated with Lewis y/IgM/CIC. By CASA, 9 samples with MUC1 values above the cut off were selected and IP was performed, followed by SDS-PAGE and Western blot; bands at 200 kDa were obtained with each MAb in all the samples. By IHC, with C14 MAb, 47.5%, 31% and 35% of malignant, benign and normal samples, respectively, showed positive reaction while all the samples were positive with anti-MUC1 MAb; in both cases, with a different pattern of expression between malignant and non malignant samples.

**Conclusion:**

Our findings support that in breast cancer there was a limited humoral immune response through Lewis y/IgM/CIC levels detection which correlated with MUC1/IgM/CIC. We also found that Lewis y might be part of circulating MUC1 glycoform structure and also that Lewis y/CIC did not correlate with Lewis y expression.

## Background

Worldwide, breast cancer is the most common cause of mortality by cancer in female population (GLOBOCAN, 2002, IARC). In order to decrease mortality and to improve treatment, prevention and early detection biomarkers are object of study. In this sense, it is very important to increase knowledge about tumor biology, which includes studies on risk factors, tumor development, dissemination and metastasis.

There is sufficient evidence that blood group related Lewis antigens are tumor-associated molecules [1]. Changes in the structure of glycan chains covalently attached to glycoproteins and glycolipids are a common feature of progression to malignancy [2]. In O-linked glycosylation, the glycans are added to serine and threonine hydroxyl groups. Initiation of O-glycosylation in the mammary gland begins in the Golgi apparatus, is catalysed by a family of enzymes which transfer N-acetylgalactosamine (GalNAc) from UDP-GalNAc (UDP-GalNAc polypeptide glycosyltransferases) to selected serine or threonine residues in protein chain [3]. After the addition of GalNAc, various core structures are formed by the addition of different sugars. The terminal epitopes of the O-glycans on mucins are probably the most important determining whether the molecule plays a role in cell adhesion phenomena. The epitopes recognized by antibodies related to the ABO and Lewis blood group antigens are found in this region. Terminal sugars added in alpha linkage include sialic acid, fucose, galactose, GalNAc and N-acetylglucosamine (GlcNAc). Some sulphation of sugars in terminal structures may also occur [4].

Lewis y antigen is a difucosylated oligosaccharide with the chemical structure:



This molecule is expressed predominately during embryogenesis while in adults, expression is restricted to granulocytes and epithelial surface [5].

Lewis y and Lewis b antigens are over-expressed by breast, lung, colon, pancreas, prostate and ovarian cancers, either at the plasma membrane as a glycolipid or linked to surface receptors such as Erb-B family receptors [1]. Sialyl-Lewis x and sialyl-Lewis a are complex carbohydrates which have been also found in breast carcinomas [6].

Breast cancer cell glycans changes are related to glycoprotein antigenic differences between carcinoma and normal mammary gland cells [7]. This phenomenon has been extensively studied on MUC1 mucin where the aberrant glycosylation found in tumor cells indicates the appearance of novel glycan epitopes (e.g. STn) as well as the unmasking of peptide sequences (rev. in [4]). Lewis y oligosaccharides may be part of mucin glycoproteins, which have characteristic core peptide structures [8]. MUC1, which is overexpressed in breast cancer, may contain Lewis y. This mucin has been involved in immune regulation, cell signaling, inhibition of cell-cell and cell-matrix adhesion [9]. Glycan changes may be important to the induction of a humoral response [10]. Cell-surface antigens (mainly carbohydrate antigens) have proved to be unexpectedly potent targets for immune recognition and attack against human cancers [11].

In a variety of different clinical settings, correlation of antibodies naturally acquired or vaccine induced with prognosis improvement is one of the bases for cancer vaccines designed primarily for antibody induction [12].

In tumor patients sera, it has been frequently found the occurrence of variation in circulating immune complexes' (CIC) levels [13-16]. Although the overall composition of CIC varies quantitatively even for patients with the same malignancy, MUC1 has been described as a part of CIC associated with cancer including breast carcinoma [13,16,17]. It has been postulated that CIC may play a protective [15] as well as an impaired [14,18,19] function.

In this sense, the first aim of this research in breast cancer samples was to evaluate the presence of Lewis y and MUC1 in circulating immune complexes (Lewis y/CIC and MUC1/CIC, respectively) and their correlation in order to investigate their involvement in natural humoral immune response.

The second aim of this study was to analyze the possible presence of Lewis y in carbohydrate chains of tumoral MUC1 glycoprotein isolated from serum.

The third aim was to correlate serum and tissue parameters considered.

## Materials and methods

### Samples

One hundred and forty six pretreatment serum and tissue samples proceeding from 76 breast cancer patients, 34 benign breast disease patients and 36 from women without disease were processed. Breast cancer samples were 82% ductal, 13% lobular and 5% mucinous. Disease staging was: 13% *in situ *carcinoma, 30% stage I, 34% stage II, 20% stage III and 3% stage IV.

Patients mean age was 55, with a range from 28 to 85 years old.

Breast cancer samples were obtained during tumor resection surgery and control breast tissue samples from breast reduction surgery performed since 2005 to 2007 at different hospitals related to the National University of La Plata, La Plata, Buenos Aires, Argentina. Serum samples were aliquoted and stored at -70°C until analyzed.

Experiments were done according to the Helsinki Declaration. Informed consent was obtained from all women included in this study. This research was approved by the Local Human Investigation Committee, Faculty of Medical Sciences, National University of La Plata, Argentina.

### Monoclonal Antibodies (MAbs)

The following MAbs were assayed: C595, SM3, HMFG1 MAb, directed against different epitopes of a sequence of 20 repeated aminoacids in tandem: variable number of tandem repeat (VNTR) in the MUC1 protein core [16,20] and C14 (IgM) MAb, an anti-Lewis y carbohydrate [21].

## Methods

### ELISA (enzyme linked immunosorbent assay) for the detection of circulating immune complexes carrying the Lewis y carbohydrate (Lewis y/CIC)

Lewis y/CIC levels were measured by an ELISA method employing C14 MAb. One hundred μl of 1/100 C14 MAb diluted in buffer carbonate/bicarbonate pH 9.6 were adsorbed in each well of 96 wells ELISA microplates (Falcon 3912, Microtest III, Becton Dickinson Labware, Oxnard, USA). After overnight incubation at 4°C, several washes with sodium phosphate buffer/0.1% Tween 20 (PBST) were done. In each well, 200 μl of blocking buffer (1%BSA/PBS) were added and plates were incubated at 37°C for 3 h. One hundred μl of 1/20 serum samples diluted in PBS were applied by triplicate and incubated overnight at 4°C with the absorbed MAb. Then, plates were washed with PBST and 1% Triton X-100/PBS; after that, 1/2000 anti-human IgM or 1/3000 anti-human IgG horseradish peroxidase conjugates (Dakopatts, Dako Corporation, Copenhagen, Denmark) were added and incubated at 4°C for 2 h. Then, freshly prepared 2,2'-azino-bis (3-ethylbenzothiazoline)-6-sulphonic acid, (ABTS, SIGMA, St. Louis, MO, USA) as substrate in sodium citrate buffer (0.1 M citric acid, 0.2 M PO_4_HNa_2_·12H_2_O), pH 5.0 and 30% H_2_O_2 _was added.

Results were expressed as optical density (OD) units at 405 nm. The intra-assay coefficient of variation (CV) obtained was 3.0% while the inter-assay CV obtained was 10.6%.

### ELISA for the detection of MUC1 circulating immune complexes (MUC1/CIC)

The technique was developed according to previous reports [16]. Briefly, MUC1-CIC were measured by an ELISA test employing a MUC1-specific murine MAb to capture this glycoprotein: C595 (IgG3, anti-RPAP). The MAb was adsorbed in Falcon plates (Falcon 3912 Microtest III, Becton Dickinson Labware, Oxnard); 100 μl per well of human serum previously diluted 1:20 in PBS were applied in duplicate. After incubation and carefully washed, 100 μl of diluted rabbit anti-human IgM or IgG immunoglobulins, horseradish peroxidase conjugates (Dakopatts, Dako Corporation, Copenhagen, Denmark) were added; afterwards, plates were carefully rinsed and, 100 μl per well of freshly prepared 2,2'-azinobis(3-ethylbenzothiazoline)-6-sulphonic acid, ABTS (Sigma Chemical Co., MO, USA) in sodium citrate buffer (0.1 M citric acid, 0.2 M PO_4_HNa_2_·12H_2_O), pH 5.0 and 30% H_2_O_2 _was added.

For each serum sample, results were expressed as a mean difference from OD at 405 nm of MAb coated wells; OD obtained without serum was subtracted from mean OD of the sample wells.

### MUC1 detection by CASA test

MUC1 serum levels were measured by a commercial CASA test using a dual determinate ELISA (Medical Innovations Limited, Artarmon, Australia). All the steps of the CASA test were made according to the manufacturers' instructions. The working range was between 2 and 64 units/ml; samples that exceeded 64 units/ml were diluted 1/5 in negative control and re-assayed. This test utilizes MAbs BC2 (IgG) and BC3 (IgM), both detecting the peptide epitope APDTR on the VNTR region of the protein core of the MUC1 mucin; the cut off level was 2 units/ml.

### Immunoprecipitation (IP) of MUC1 from serum samples

Five hundred μl of serum were added to 50 μl of protein A-Sepharose CL-4B (SIGMA, St. Louis, MO, USA); incubated on ice for 30–60 min and spun down at 10000 × g at 4°C for 10 min. Precleared serum was incubated at 4°C for 1 h with 10 μl of HMFG1 MAb. Fifty μl protein A-Sepharose CL-4B was added to immune complexes and shook on a rotator at 4°C for 1 h. After spinning, the supernatant was removed and the pellet was washed with lysis buffer (1% NP40, 1 mM phenyl methyl sulphonyl fluoride, 150 mM NaCl, 50 mM Tris-HCl, pH 8.0) (SIGMA, St. Louis, MO, USA). Then, 50 μl of Laemmli buffer (2% SDS, 5% 2-mercapoethanol, 10% glycerol) was added and heated to 90–100°C for 10 min. After spun down, the supernatant was loaded on the gel for SDS-PAGE analysis.

### SDS-PAGE and Western blot (WB) of IP

Supernatants were analyzed under reducing conditions in SDS-PAGE in a discontinuous buffer system according to Laemmli [22]. After electrophoresis, gels were either stained with Coomassie blue (SIGMA, St. Louis, MO, USA) or they were electrophoretically transferred to nitrocellulose membranes [23] which were blocked with PBS/5% skimmed milk (blocking buffer). After washing with PBST, sheets were incubated with either HMFG1 MAb or C14 MAb diluted in blocking buffer. HMFG1 MAb was employed undiluted while C14 MAb was diluted 1/100 in blocking buffer. Sheets were incubated overnight at 4°C and rinsed with PBST buffer. A final incubation with 1/400 peroxidase-conjugated anti-human immunoglobulins was performed according to the manufacturer's instructions (SIGMA, St. Louis, MO, USA). Nitrocellulose sheets were developed with 3,3'-diaminodiazobenzidine in PBST containing 30% H_2_O_2_.

### Immunohistochemistry (IHC)

In all samples, the technique was performed following standard procedures: paraffin embedded specimens were treated with 10 mM sodium citrate buffer pH: 6.0 at 100°C for 10 min and incubated overnight at 4°C with mouse anti-Lewis y and anti-MUC1 MAbs. Negative controls were incubated with PBS instead of MAb. A final incubation with 1/400 peroxidase-conjugated goat anti-mouse IgM immunoglobulins (SIGMA, St. Louis, MO, USA) was performed. The chromogen employed was 3,3'-diaminodiazobenzidine (SIGMA, St. Louis, MO, USA) in 1%BSA/PBS containing 30% H_2_O_2_.

Sections were examined by light microscopy and the antibody staining patterns were scored in a semiquantitative manner. Staining intensity was graded as negative (-), low (+), moderate (++), or strong (+++). The number of optical fields in a specimen that were positively stained was expressed as a percentage of the total number of optical fields containing tissue. The staining of cytoplasm, plasma membrane and nucleus was evaluated; cells were considered positive when at least one of these components was stained. The pattern of reaction was classified as linear (membrane reaction), cytoplasmic, or mixed (cytoplasmic and membrane) and the positive reaction in gland lumen content was identified as cellular debris or secretion. Apical and non-apical reactions were also considered [24].

### Statistical analysis

Normality of Lewis y/CIC values and staining intensity were tested by Shapiro Wilk's and normalized.

In the case of Lewis y/CIC levels, groups were compared by one way ANOVA followed by Tukey HSD for an unequal number of cases post hoc comparisons (p < 0.05).

Statistical differences for immunohistochemical results were evaluated by the Chi square test. A Principal component analysis (PCA) was performed among CIC and classical correlation among transformed data was performed (p < 0.05).

## Results

### Detection of Lewis y/CIC

**Figure 1:**
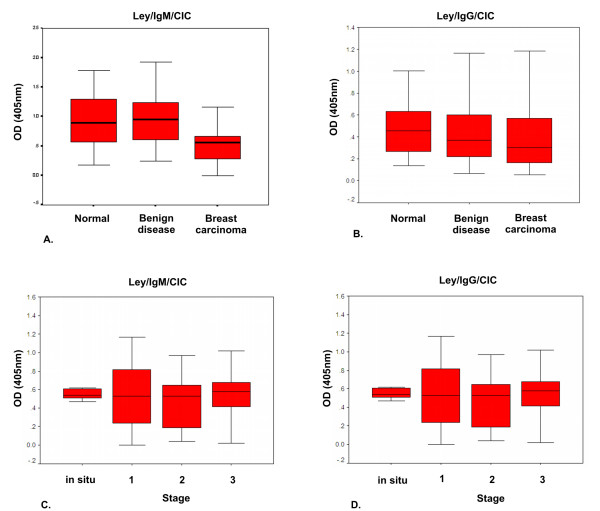
**A-D Box-plots represent median values and interquartile ranges of Le^y^/IgM/CIC (A, C) and Le^y^/IgG/CIC (B, D) measured by ELISA in normal, benign and malignant breast samples (A, B), and in different stages (C, D) of breast cancer**. Results are expressed as OD units (405 nm).

An ELISA method was developed to detect Lewis y/CIC; C14 MAb anti-Lewis y was used to capture immune complexes present in serum samples and they were detected through a peroxidase-conjugated anti-human IgM or IgG. The reaction was revealed with ABTS as substrate and OD at 405 nm was measured. Lewis y/IgM/CIC mean values obtained were the following: 0.525 ± 0.304 (mean ± SD) OD units for breast cancer samples; 0.968 ± 0.482 for benign disease and 0.928 ± 0.447 for normal samples. By ANOVA, standardized Lewis y/IgM/CIC levels from cancer serum samples were significantly lower than normal and benign levels (p < 0.05), which did not differ between them (Fig. 1A).

Lewis y/IgG/CIC OD mean values were: 0.418 ± 0.318; 0.461 ± 0.321 and 0.485 ± 0.267 for breast cancer, benign and normal samples, respectively. No differences were found among groups (Fig. 1B). There was no difference in Lewis y/CIC values among breast cancer types.

Differences among breast cancer stages were studied by ANOVA on standardized data and any difference was found neither for Lewis y/IgM/CIC nor for Lewis y/IgG/CIC levels (Fig. 1C and 1D, respectively).

### Detection of MUC1/CIC

MUC1/IgM/CIC mean values obtained were the following: 0.320 ± 0.253 (mean ± SD) OD units for breast cancer samples; 0.453 ± 0.473 for benign disease and 0.406 ± 0.302 for normal samples.

MUC1/IgG/CIC OD mean values were 0.763 ± 0.276; 0.758 ± 0.251 and 0.831 ± 0.359 for breast cancer, benign and normal samples, respectively. No differences were found among groups. By ANOVA, standardized MUC1/CIC levels did not differ among groups.

### Immunoprecipitation (IP), SDS-PAGE and WB

MUC1 IP was performed in nine serum samples from patients with malignant and benign breast diseases as well as normal females with CASA values above the cut-off level (2 Units/ml).

**Figure 2:**
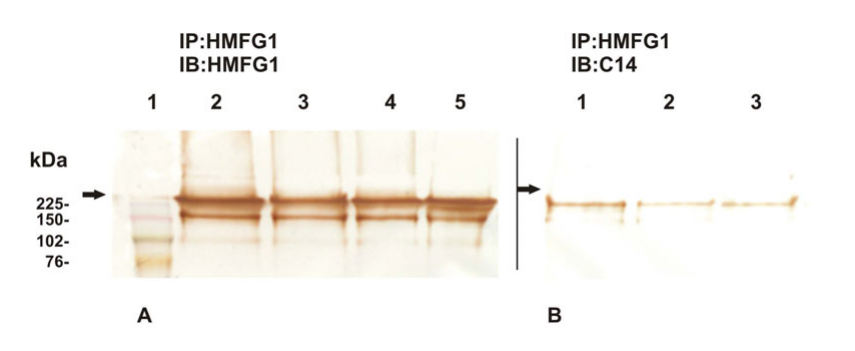
**A & B: (A) Immunoblotting (IB) of samples obtained by immunoprecipitation (IP) with HMFG1 MAb from sera and incubated with HMFG1; 1: MW Standard, 2: normal sample, 3: benign disease sample; 4: breast cancer sample; 5: Standard of MUC1 (32 U/ml)**. (B) IB of samples obtained by IP with HMFG1 MAb from sera and incubated with C14; 1: normal sample, 2: benign disease sample; 3: breast cancer sample. Bands at 200 kDa are shown with each MAb. The arrows indicate the start of the resolving gel.

In order to isolate MUC1 from sera, pellets obtained by IP using HMFG1 MAb were treated with lysis and Laemmli's buffer. All samples and supernatants obtained were analyzed by SDS-PAGE and WB. Blotting sheets were incubated with C14 MAb and HMFG1 MAb; the latter was employed to validate IP results. With each MAb, bands at 200 kDa were identified in all selected samples indicating that MUC1 should contain Lewis y carbohydrate in its structure. Fig. 2A and 2B show the band obtained from a normal, a benign and a breast cancer sample when the membranes were incubated with HMFG1 and C14, respectively. In Fig. 2A it was included a standard of 32 Units/ml of MUC1 provided by CASA test in order to verify that MUC1 was well obtained after IP.

### Lewis y expression by IHC

All samples were analyzed (n = 146); percentages of positive reaction with C14 MAb in relation to total were as follows: 47.5% of tumor samples, 31% of benign samples and 35% of normal samples. Frequency analysis was performed; groups were compared by the Chi square test and non significant difference was found (p > 0.05).

According to tumor stages the percentages of positivity (positive samples/total samples of each stage) analyzed were: 20% of *in situ*, 36% of stage I; 32% of stage II and 47% of stage III; 33% of stage IV and non significant differences were found (p > 0.05).

**Figure 3:**
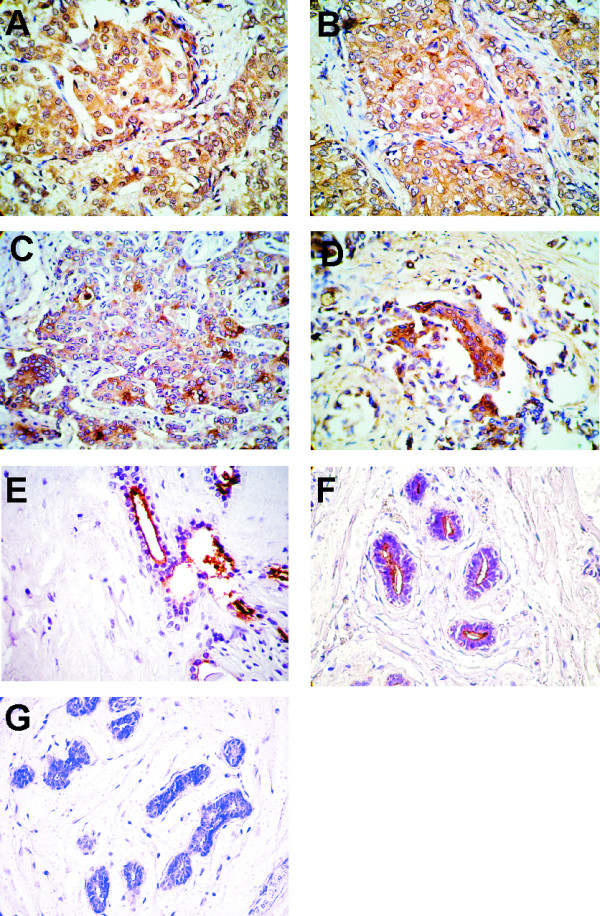
**Microphotographs of IHC are shown (×400)**. Ductal breast carcinoma sections at stages (A) I, (B) II, (C) III and (D) IV incubated with C14 anti-Lewis y MAb. A mainly non-apical cytoplasmic positive reaction is shown in all samples. (E) A benign and (F) a normal breast samples with an apical and linear pattern are shown. (G) A normal sample which did not react with C14 is depicted.

Although there was any statistical difference, the pattern of expression differed between malignant and non malignant samples. In cancer specimens, a mixed pattern (cytoplasmic and membrane) with non apical reactivity was more frequently detected at different stages (Fig. 3A–D) compared with the apical membrane pattern found in benign (Fig. 3E) as well as in normal samples (Fig. 3F). In malignant specimens, variation of Lewis y expression was a common feature. In several tumors, diffuse and moderate or intense staining was mainly restricted to non apical cytoplasm; some samples showed a cytoplasmic reaction with a strong intensity and a granular pattern. Other specimens had a strong reaction limited to the apical part of the cells (cytoplasm and membrane) in lining glands and also in lumen content. In some tumor sections, an intense staining at the apical blebs was found. No nuclear staining was observed. Fig. 3G shows a normal sample which did not react with C14 MAb.

### MUC1 expression by IHC

**Figure 4:**
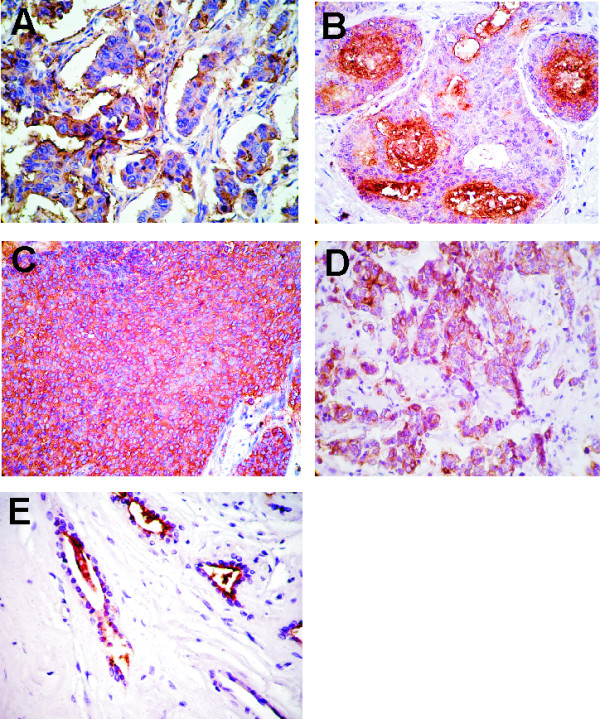
**Microphotographs of IHC of ductal breast carcinoma samples at different stages are shown (×400)**. (A) Stage I, (B) II, (C) III and (D) IV sections incubated with anti-MUC1 MAbs reacted with a non-apical mainly mixed pattern; in (E) a benign sample shows an apical linear positive reaction; content of a ductal lumen is also stained.

All samples were analyzed (n = 146); most malignant, benign and normal breast samples expressed MUC1; despite the stage of the disease, malignant tumors showed reactivity mainly at the cytoplasm with a non-apical pattern although frequently plasmatic membranes also showed staining (mixed pattern) (Fig. 4A–D). The intensity of the reaction varied from moderate to strong. As it was expected, benign and normal samples mainly showed an apical and linear pattern. In Fig. 4E a positive reaction of a benign breast disease sample is also shown.

### Analysis of correlations

In cancer and benign samples, considering intensity of the IHC reaction versus Lewis y/CIC levels, no significant correlation was found. Lewis y/IgM/CIC and Lewis y/IgG/CIC values did not correlate as well. In benign samples, although there was not any statistical significance, Lewis y/IgG/CIC levels showed a decrease tendency to decrease while intensity increased (R^2 ^= -0.66).

Normal samples showed a high and significant correlation among staining intensity versus Lewis y/IgM/CIC and Lewis y/IgG/CIC levels (R^2 ^= 0.885 and 0.967, respectively); in the case of Lewis y/IgM/CIC, a poor but significant correlation with Lewis y/IgG/CIC was found (R^2 ^= 0.326, p < 0.05).

**Figure 5:**
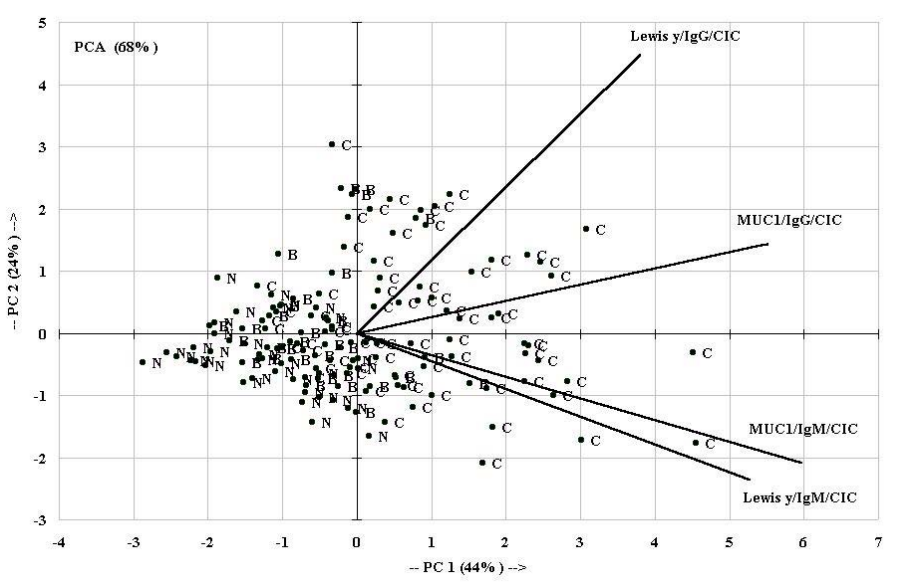
**Principal Component Analysis (PCA) was performed employing Lewis y/IgM/CIC, Lewis y/IgG/CIC, MUC1/IgG/CIC and MUC1/IgM/CIC**. First and second component explained 68% of data variability; normal samples and benign samples appeared grouped (PC1 (-)) and separated from cancer samples which remained spread. All variables weighed similar in the model, Lewis y/IgM/CIC, MUC1/IgG/CIC and MUC1/IgM/CIC predominated PC1 (+) while Lewis y/IgG/CIC was shared between PC1(+) and PC2(+). Rays and circles represent CIC analyzed and cases, respectively. C: cancer, B: benign, N: normal.

In order to explore data, PCA was performed employing Lewis y/IgM/CIC, Lewis y/IgG/CIC, MUC1/IgG/CIC and MUC1/IgM/CIC. First and second component explained 68% of data variability; normal samples and benign samples appeared grouped (PC1 (-)) and separated from cancer samples which remained spread. All variables weighed similar in the model, Lewis y/IgM/CIC, MUC1/IgG/CIC and MUC1/IgM/CIC predominated PC1 (+) while Lewis y/IgG/CIC was shared between PC1(+) and PC2(+) (Fig. 5).

Classical multiple correlations (p < 0.05) are shown in Table 1; in consequence, normal samples appeared grouped.

**Table 1:** Spearman correlation coefficients among CIC levels

	Le y/IgM/CIC	Le y/IgG/CIC	MUC1/IgM/CIC	MUC1/IgG/CIC
Le y/IgM/CIC	**1**	**0.2147**	**0.4038**	**0.2847**
Le y/IgG/CIC	**0.2147**	**1**	0.0739	**0.3362**
MUC1/IgM/CIC	**0.4038**	0.0739	**1**	**0.5118**
MUC1/IgG/CIC	**0.2847**	**0.3362**	**0.5118**	**1**

Bold letters indicate significant correlations.

Lewis y and MUC1 expression as well as CIC levels did not show any significant difference among tumor stages.

## Discussion

It has been proved that MUC1 is extremely useful for glycosylation studies because changes in the expression and activity of specific glycosyltransferases have been related to changes in the *O*-glycan structures expressed by MUC1 [4]. In a previous report we found that Lewis × antigen was highly expressed by normal epithelial tissues of mammary gland and digestive tract [24]. In order to continue the study of blood group related Lewis antigen involvement in breast cancer, we have focused this research on the difucosylated Lewis y antigen; this carbohydrate specifically belongs to the ABH Lewis blood group family which is overexpressed on the majority of carcinomas including ovary, pancreas, prostate, breast, colon and non small cell lung cancers [25-27].

We performed immunoprecipitation of MUC1 from breast cancer, benign and normal serum samples with HMFG1 MAb, directed against MUC1 peptide core (DTR) and isolated the glycoprotein. SDS-PAGE and Western blot assays were performed with the samples obtained by IP; nitrocellulose membrane incubation with C14 MAb showed the same MW band as incubation with HMFG1 MAb in breast cancer, benign and normal samples. These results indicate that Lewis y could be involved in MUC1 structure. Sikut et al. found that sialyl Lewis a and sialyl Lewis × epitopes were attached to MUC1 in breast cancer patients serum samples [28].

During many years, the functions of Lewis y were mostly unknown although it was described as a differentiation and onco-developmental antigen [8]. Basu et al. found that in colon and vulval carcinoma cell lines, sialylated Lewis a and Lewis y were present in the EGF receptor glycoprotein [29].

In the last decades, further information was achieved; in breast cancer cell lines, Hellström et al. probed that MAbs reactive against Lewis y could be internalized and mediated tumor cell killing by antibody-dependent cellular cytotoxicity (ADCC) and complement dependent cytotoxicity (CDC) [30]. Furthermore, sialylated Lewis a and Lewis y were related with apoptosis; in this sense, Rapoport and Le Pendu found in colon carcinoma cell lines such as HT29 in which apoptosis was induced by UV irradiation, TNFα and anti-Fas, a major decrease of this antigen as well as Lewis x [31]. On the other hand, in Jurkat human T cell line, the expression of Lewis x and Lewis y was enhanced in the cell surface during apoptosis induced by different agents including anti-Fas antibody [32].

Lewis y is attached to components of the CD66 cluster which is a member of the carcinoembryonic antigen (CEA) family and of the immunoglobulin superfamily. The activation-increase in Lewis y attached to CD66 adhesion molecules implicates a role of the Lewis y determinant in cytoadhesive properties of granulocytes on trafficking and inflammatory responses [5,33].

In cancer cells, Miyake et al have observed that MAbs which bind to Lewis y antigen, although cross-reacted with H and Lewis b, inhibited cell motility and tumor cell metastasis [34].

We employed C14 MAb which recognizes circulating antigen in serum and tumor sections of patients bearing a variety of malignancies [35]. In this study by IHC, with this MAb, we found a positive reaction in 47.5% of breast tumor samples, showing a different pattern of expression among malignant, benign and normal samples; nevertheless; no statistically significant difference in percentage of expression was found. In cancer samples, we did not find any significant difference among different stages. Our results are in agreement with Madjd et al since they found that Le^y/b ^is expressed in 44% of breast cancer samples, employing SC101 MAb although this MAb reacts with both Lewis y and Lewis b [1].

On the other hand, as it was not surprising, MUC1 was detected in all samples employing many anti-MUC1 antibodies (16); in consequence, correlation analysis was not necessary.

Klinger et al confirmed that the majority of cancer cells derived from epithelial tissue express Lewis y type difucosylated oligosaccharides on their plasma membranes; they have also found that ABL 364 MAb against this carbohydrate which is present in erbB receptor side chains are capable of inhibiting erbB receptor mediated signaling [36]. Other authors found a novel function for soluble Le^y^/H as an endothelial-selective and cytokine inducible as well as a potent angiogenic mediator in both in vitro and in vivo bioassays [37].

Cancer antigens expressed at the cell surface are generally glycolipids or glycoproteins [12,38] which may express in their molecules blood group related Lewis antigens [2]. The non appropriate biosynthesis or processing of carbohydrate structures may contribute to the disordered behaviour of tumor cells [39]. Lewis y carbohydrate may participate in natural humoral immune response; antibodies are ideally suited for eradicating pathogens from bloodstream and early tissue invasion. With regard to cancer cells, passively administered and vaccine induced antibodies have accomplished this concept, limiting tumor cells and systemic or intraperitoneal micrometastases in a variety of preclinical models [12]. Many protocols developing anti-Lewis y vaccines have been performed [12,40,41].

In this report, we found that Lewis y/IgM/CIC levels correlated with Lewis y/IgG/CIC levels and MUC1/CIC (IgG and IgM) levels and also that Lewis y/IgG/CIC levels correlated with MUC1/IgG/CIC levels. These correlations may be related with the fact that MUC1 may be a carrier of Lewis y epitope. Von Mensdörff-Pouilly et al [42] found that naturally occurring MUC1 antibodies seem to check disease spread in breast cancer patients, possibly by destroying blood-borne isolated disseminated tumor cells (micrometastases) which eventually could lead to metastatic disease and death. Silk et al found significantly higher anti-MUC1 IgG levels in abnormal versus normal colorectal location [43].

Correlation of antibodies, naturally acquired or vaccine-induced, with improved prognosis in a variety of different clinical settings is one of the bases for cancer vaccines designed for antibody induction. It is essential to ensure that immune responses against tumor antigens can destroy tumor cells but not normal ones. An important immune response against a tumor specific antigen would be irrelevant if a tumor cell mutates in such a way that it no longer expressed its specific antigen avoiding cells destruction by the immune system [44]. Therefore, it is remarkably outstanding to study the natural humoral immune response through immune complexes detection. With the aim of enhancing immune response in breast cancer patients, vaccines constructed with glycolipids or glycoproteins derivatives as immunogens are being developed.

## Conclusion

By IHC, tumor and tissue Lewis y and MUC1 expression was evaluated; although we did not find any statistically significant difference among malignant, benign and normal samples, the pattern of expression differed. Besides, no correlation between clinical pathological parameters (age, type, stage or grade) and IHC expression was found.

On the other hand, humoral immune response was studied measuring Lewis y/IgM/CIC levels and a statistically significant difference among breast cancer serum samples versus normal and benign specimens was found, being lower in cancer samples.

Our findings also support that, in breast cancer, Lewis y may be part of circulating MUC1 glycoform structure and that Lewis y/CIC do not correlate with Lewis y expression. This lack of correlation may be related to a limited humoral immune response against these molecules in cancer patients which could be due to the escape from the immunosurveillance of the host.

## Abbreviations

CIC: Circulating immune complexes; MUC1: Mucin 1; MAb: Monoclonal antibody; PBS: sodium phosphate buffer; PBST: sodium phosphate buffer/0.1% Tween 20; ELISA: enzyme linked immunosorbent assay; SDS-PAGE: sodium dodecyl sulfate polyacrylamide gel electrophoresis; WB: western blot; IP: Immunoprecipitation

## Competing interests

The authors declare that they have no competing interests.

## Authors' contributions

MIL designed and constructed the ELISA and performed the manuscript. IP and WB were done by MIL and EL. Immunohistochemical staining was performed by MIL and MC. Statistical analysis was done by MIL and SD. MIL and EL assisted with design and interpretation of the study. AB, AC and FT provided the cancer samples. MVC observed and evaluated the IHC slides and obtained the microphotographs. Histopathological diagnosis was performed by AS-E. Overall supervision of the scientific research was completed by AS-E and MVC. 
